# Enzymatic activities and arbuscular mycorrhizal colonization of *Plantago lanceolata* and *Plantago major* in a soil root zone under heavy metal stress

**DOI:** 10.1007/s11356-015-5695-9

**Published:** 2015-11-04

**Authors:** Ewa Gucwa-Przepióra, Aleksandra Nadgórska-Socha, Barbara Fojcik, Damian Chmura

**Affiliations:** Department of Botany and Nature Protection, University of Silesia, Jagiellońska 28, 40-032 Katowice, Poland; Department of Ecology, University of Silesia, Bankowa 9, 40-007 Katowice, Poland; Institute of Engineering and Environmental Protection, University of Bielsko-Biała, Willowa 2, 43-309 Bielsko-Biała, Poland

**Keywords:** *Plantago*, Heavy metals, Soil enzymes, Mycorrhiza

## Abstract

The objectives of the present field study were to examine the soil enzyme activities in the soil root zones of *Plantago lanceolata* and *Plantago major* in different heavy metal contaminated stands. Moreover, the investigations concerned the intensity of root endophytic colonization and metal bioaccumulation in roots and shoots. The investigated *Plantago* species exhibited an excluder strategy, accumulating higher metal content in the roots than in the shoots. The heavy metal accumulation levels found in the two plantain species in this study were comparable to other plants suggested as phytostabilizers; therefore, the selected *Plantago* species may be applied in the phytostabilization of heavy metal contaminated areas. The lower level of soil enzymes (dehydrogenase, urease, acid, and alkaline phosphatase) as well as the higher bioavailability of metals in the root zone soil of the two plantain species were found in an area affected by smelting activity, where organic matter content in the soil was also the smallest. Mycorrhizal colonization on both species in the contaminated area was similar to colonization in non-contaminated stands. However, the lowest arbuscule occurrence and an absence of dark septate endophytes were found in the area affected by the smelting activity. It corresponded with the lowest plant cover observed in this stand. The assessment of enzyme activity, mycorrhizal colonization, and the chemical and physical properties of soils proved to be sensitive to differences between sites and between *Plantago* species.

## Introduction

The trace metals released into the environment through human activities (such as smelting, energy and fuel production, power transmission, agriculture, foundries, and especially waste deposits from Zn-Pb ore mining) have been reported to be dangerous to the ecosystems as well as their inhabitants—human beings (Nouri et al. 2009; Olowoyo et al. 2012; Wójcik et al. 2014). This type of environmental pollution may be indicated by some plants; thanks to their ability to absorb and accumulate metals with different accumulation strategies (Markert et al. 2008; Bekteshi and Bara 2013); this ability of autochthonous plant cover is also commonly used in the remediation of mine tailings (Olowoyo et al. 2012; Wójcik et al. 2014). Knowledge about the capabilities of plant species naturally occurring in contaminated areas to absorb and transport metals will provide information for choosing appropriate plants for phytoremediation purposes.

The two plantain species: *Plantago lanceolata* and *Plantago major* investigated in the study are representatives of the ruderals in natural vegetation in Poland. Ruderals are widely spread and recommended as bioaccumulation indicators with the ability to accumulate metals in large quantities without visible damage. The two plantain species are characterized by a tolerance to metal contaminants and other adverse site conditions connected with antioxidant defense, as discussed in a previous study (Nadgórska-Socha et al. 2013). *P. lanceolata* is still an object of many studies as an indicator of metals in shoots and in the soil (Tamás and Kovács 2005; Dimitrova and Yurukova 2005). It is strongly colonized by AM fungi, and mycorrhizal colonization of this plant has been proposed as a useful tool in the bioindication of soil quality and effectiveness of restoration processes (Orłowska et al. 2002, 2005, 2012). The other plantain species, *P. major*, has been postulated as a bioindicator, e.g., in a study of the Region of Durrës (Bekteshi and Bara 2013), and used in determining the efficacy of heavy metals bioaccumulation and translocation potential at a heavy traffic affected soil site (Galal and Shehata 2015). Despite the research on the accumulation ability of selected heavy metals in *Cardaminopsis arenosa*, *P. lanceolata*, and *P. major* leaves and antioxidative defense responses in metalliferous soil (Nadgórska-Socha et al. 2013; 2015), there is insufficient data on AM colonization by excluders—plantain species in field studies, especially in contaminated areas. The development of plant cover is important for the restoration of the physical, chemical, and biological properties of contaminated soils, which naturally reduce the mobility and bioavailability of heavy metals via sorption, precipitation, and complexation reactions (Pérez de Mora et al. 2005). Plants are additionally significant for soil biota through influencing the quality and quantity of organic substrates in the soil. The plant cover and organic matter content also play important roles in determining soil enzyme activity as extracellular enzymes are derived mainly from soil microorganisms, plant roots, and soil animals. Soil phosphatases are enzymes with a relatively broad specificity, capable of hydrolyzing various organic phosphate esters, and are involved in the P cycle (Dick 1997; Yang et al. 2010; Fernández et al. 2012). Dehydrogenase activity reflects the total oxidative activities of soil microflora, important in oxidizing soil organic matter (Dick 1997), and active inside the viable cells. The activity of dehydrogenase is similar to the number of active microorganisms. This enzyme has been considered as a sensitive indicator of soil quality and a valid biomarker to indicate changes in total microbial activity due to changes in soil management (Dick 1997; Kieliszewska-Rokicka 2001; Nadgórska-Socha et al. 2006; Fernández et al. 2012). Urease catalyzes the hydrolysis of urea into ammonia or ammonium ion depending on soil pH and carbon dioxide. An extracellular enzyme is affected by plant type or species combinations (Yang et al. 2007, 2010).

Heavy metals can affect microbial processes and decrease the number and activity of soil microorganisms among these arbuscular mycorrhizal fungi. However, long-term heavy metal effects can increase its tolerance to metals. Arbuscular mycorrhiza (AM) is the most ancestral and common type of mycorrhizal symbiosis (Brundrett 2002), in which the fungal hyphae penetrate the cortical cell wall of the host plant root. It is characterized by arbuscules and vesicles formed by the aseptate, obligate symbiotic fungi of the phylum Glomeromycota (Schüßler et al. 2001), which can play an important role in heavy metal stress mitigation as well as in the restoration of contaminated ecosystems (Gucwa-Przepióra and Turnau 2001; Pérez de Mora et al. 2005; Gucwa-Przepióra et al. 2007; Yang et al. 2007). Moreover, there is evidence that AM fungi can benefit plant nutrition and enhance plant tolerance to heavy metal pollution, in part by immobilization of metals within or near the root and reducing their translocation to the shoot (Carrasco et al. 2011).

Dark septate root endophytes (DSE) are an artificial assemblage of fungi that have darkly pigmented septate hyphae and are frequent intracellular root associates of plants (Piercey et al. 2004). They colonize the cortical cells and intercellular regions of roots and form densely septated intracellular structures called microsclerotia (Jumpponen and Trappe 1998). Similarly to AM fungi, it might be possible that DSE colonization plays an important role in improving plant fitness (Addy et al. 2005; Likar and Regvar 2013).

When assessing the toxicity of contaminated soils, both heavy metal concentration and availability must first be determined. So far, information linking heavy metal bioavailability and toxicological response in soils has been very limited (Wang et al. 2007). In this work, we reported the results of a study carried out on *P. lanceolata* and *P. major* growing on heavy metal contaminated soils and on the heavy metal bioaccumulation and translocation efficiency. The aim of this study was to examine soil enzyme activity in soil root zones with different heavy metal contamination and the relationships between soil enzyme activity and selected heavy metal bioavailability. The investigations were undertaken to study the intensity of root endophytic colonization (AM and DSE) and metal bioaccumulation in roots and shoots. Furthermore, the application of soil enzyme activity and mycorrhizal colonization indices in bioindication field studies was discussed.

## Materials and methods

### Study area

The study was performed in three areas situated in the Śląskie and Małopolskie provinces in southern Poland. Two sites were contaminated with heavy metals: the vicinity of a former metal smelting plant “Szopienice” (Sz) in Katowice 50° 15′ 29.65″ N, 19° 6′ 42.88″ E, and a zinc-lead (calamine) site in Dąbrowa Górnicza (D) 50° 18′ 58.859″ N, 19° 18′ 28.62″ E, connected with ore mining and the processing of calamine zinc ores. The third locality was a non-contaminated area in the perimeter of the Pazurek Nature Reserve in Jaroszowiec (J) near Olkusz 50° 19′ 58.74″ N, 19° 35′ 59.82″ E. The investigated stands were chosen due to the different metal content (the greatest in Dąbrowa Górnicza) and the origin of contamination in the soil (vicinity of smelter, calamine postmining area). The heavy metal content in these soils had been previously ascertained (Nadgórska-Socha et al. 2013). The estimated amounts of Cd in this study were 90.8; 301.2; 2.7 mg kg^−1^, Zn 8403; 70,446, 359 mg kg^−1^; Pb 395, 3619, 123 mg kg^−1^ in Szopienice (Sz), Dabrowa Górnicza (D) and Jaroszowiec (J), respectively.

Plant cover at the three sites, according to the Braun-Blanquet method, is given in Table 1. The analyzed areas differed in the degree of vegetation. In Dąbrowa Górnicza (D), the vegetation was almost seminatural, with a three-layered mixed forest (the first trees were planted in the 1970s). The area in the vicinity of non-ferrous metal smelter (Sz) was characterized by the sparsest plant coverage, and both in the site affected by smelting activity (Sz) and in non-contaminated area in Jaroszowiec (J) meadow plants prevailed (from the Molinio-Arrhenatheretea class).

**Table 1:** Structure of plant cover of analyzed areas

	Sz	D	J
Coverage of tree layer in %	–	50	–
Coverage of shrub layer in %	–	20	–
Coverage of herb layer in %	60	70	90
Coverage of bryophytes layer in %	4	60	3
Total number of species	21	32	18
Plant species in tree layer:			
*Acer platanoides*	–	1.1	–
*Larix decidua*	–	2.2	–
*Pinus sylvestris*	–	2.1	–
Shrub layer:			
*Acer pseudoplatanus*	–	1.1	–
*Betula pendula*	–	1.1	–
*Corylus avellana*	–	1.1	–
*Fagus sylvatica*	–	1.1	–
*Padus serotina*	–	1.1	–
*Sorbus aucuparia*	–	1.1	–
Herb layer^a^:			
*Acer platanoides*	–	1.1	–
*Agrostis capillaris*	1.2	2.2	1.2
*Arabis arenosa*	1.1	–	–
*Artemisia vulgaris*	2.1	–	+
*Capsella bursa-pastoris*	–	–	1.1
*Cerastium arvense*	–	1.2	–
*Festuca ovina*	2.2	–	–
*Galium mollugo*	–	1.1	–
*Geum urbanum*	–	2.2	–
*Hypochoeris radicata*	–	2.1	–
*Knautia arvensis*	–	2.1	–
*Lolium perenne*	1.1	–	2.2
*Plantago lanceolata*	2.2	2.1	2.2
*Plantago major*	1.1	1.1	2.2
*Poa pratensis*	–	–	2.2
*Polygonum aviculare*	1.1	–	–
*Ranunculus acris*	–	1.1	–
*Rumex acetosa*	2.1	–	–
*Silene inflata*	2.1	2.2	–
*Taraxacum officinale*	–	–	1.1
*Taraxacum officinale*	–	1.1	–
*Trifolium pratense*	–	2.2	–
*Trifolium repens*	–	–	2.2
*Viola sylvestris*	–	1.1	–
Bryophytes layer:			–
*Barbula convoluta*	–	–	1.2
*Brachythecium albicans*	–	–	1.2
*Brachythecium rutabulum*	–	1.2	–
*Bryum caespiticium*	1.2	–	–
*Calliergonella cuspidata*	–	2.3	–
*Plagiomnium cuspidatum*	–	3.3	–
*Weisia controversa*	1.3	–	–

^a^Sporadic species in herb layer (+): *Acer negundo* (Sz), *Acer pseudoplatanus* (D), *Achillea millefolium* (Sz, J), *Agropyron repens* (Sz), *Carex hirta* (S3), *Chamomilla suaveolens* (S3), *Daucus carota* (Sz), *Echium vulgare* (Sz), *Epipactis helleborine* (D), *Euphorbia cyparissias* (D), *Fraxinus pennsylvanica* (Sz), *Hieracium pilosella* (Sz), *Leontodon autumnalis* (Sz, J), *Lotus corniculatus* (Sz), *Medicago sativa* (J), *Padus serotina* (D), *Pimpinella major* (D), *Pimpinella saxifrage* (D), *Potentilla argentea* (J), *Quercus robur* (D), *Scabiosa ochroleuca* (D), *Silene nutans* (D), *Sorbus aucuparia* (D), *Veronica arvensis* (J)

### Plant and soil sampling

The research was carried out on the shoots and roots of *P. lanceolata* and *P. major* collected during the period of flowering at the end of July 2014. At every sampling site, 20 randomly chosen plant individuals and soil samples (from the depth of 0–20 cm) were collected in three replicates. The collected soil and plant samples were transferred to the laboratory. Plant samples were taken to the laboratory on ice, separated into shoots and roots, thoroughly washed with tap water to remove any substrate and dust deposits and then rinsed twice with deionized water. Soil samples were sieved through a 2-mm screen. Half of them were air dried and used for pH assessment, heavy metal content, and organic matter estimation, and the other half remained field moist to be used for soil enzyme analysis.

### Plant and soil analysis

#### Analysis of metal concentrations

Soil pH was measured using a 1:2.5 soil to water ratio. Organic matter content (expressed in %) was measured following the method by Ostrowska et al. (1991). The concentrations of Cd, Pb, Zn, Cu, Fe, Mn, K, Mg, and Ca were analyzed. The metal content of the soil (HNO_3_-extractable fraction) was estimated according to the method by Ostrowska et al. (1991) and previously described in detail (Nadgórska-Socha et al. 2013). An HNO_3_-extractable fraction from the soils was obtained by shaking a sample (10 g) with 100 ml of 2 M HNO_3_ for 1 h. The bioavailable fraction (potentially bioavailable elements) was obtained by shaking a soil sample (1:10) with 0.01 M CaCl_2_ for 2 h (Wójcik et al. 2014). The content of metals was measured in the filtered extracts using flame absorption spectrometry (​Thermo Scientific iCE 3500).

Plant samples were oven dried at 105 °C, and dry weight subsamples (0.25 g) were wet digested in HNO_3_ at 110 °C and then diluted to 25 ml with deionized water. The content of metals was measured using flame absorption spectrometry (Thermo Scientific iCE 3500). For assurance of the quality of substrate analysis, the procedures were performed for blank samples and for certified reference materials.

#### Metal accumulation efficiency

To evaluate the metal accumulation efficiency in plants, we calculated mobility ratio (MR) and translocation factor (TF). TF is the ratio of metal concentration in shoots compared to the roots. TF > 1 indicates that a given element is efficiently translocated from the roots to shoots. MR is the ratio of metal concentration in shoots compared to the soil (Serbula et al. 2012).

#### Soil enzymes and root acid phosphatase activity

The soil enzymes activity was determined in soil samples at field moisture, sieved through a 2-mm sieve and stored at 4 °C before microbial analysis. The activity of alkaline and acid phosphatase was measured according to the method of Schinner et al. (1995). The p-nitrophenol (p-NP) released by phosphomonoesterase activity was extracted and colored with sodium hydroxide and determined photometrically at 400 nm. The phosphatase activity was expressed in μg p-NP g^−1^ dm h^−1^. The urease activity estimation was based on the colorimetric determination of ammonium formation after enzymatic urea hydrolysis (10 % solution, λ—630 nm). Urease activity was expressed as μg N g^−1^ dm. Triphenyltetrazolium chloride was the substrate used for dehydrogenase activity determination. The produced triphenyl formazan (TPF) was extracted with acetone and measured photometrically at 546 nm. The dehydrogenase activity was expressed in μg TPF g^−1^ dm 16 h^−1^ (Schinner et al. 1995).

Acid phosphatase analysis was performed on fresh root samples according to Aery (2010). One gram of roots was ground in 5-ml chilled acetate buffer and centrifuged at 15,000×*g* for 10 min. Supernatants were used as the enzyme source. After 30 min, incubation at 35 °C with substrate solution (p-nitrophenyl phosphate in acetate buffer), the reaction was terminated by adding 0.1 M NaOH and absorbance was measured at 410 nm. Enzyme activity was expressed as μmol of p-NP released min^−1^ g^−1^ fw.

To compare the heavy metal effects between contaminated soils, enzymes activity ratio (ACR) in % was introduced according to Xian et al. (2015):

$$ \mathrm{A}\mathrm{C}\mathrm{R}=\left({A}_h-{A}_c\right)/{A}_c\times 100\% $$

*A*_*h*_ and *A*_*c*_ denote enzyme activity in metal polluted (Sz and D) and control soil (J).

#### Mycorrhizal studies

For the estimation of mycorrhizal development, the roots were prepared according to a modified method of Phillips and Hayman (1970). After being careful washed in tap water, the roots were softened in 7 % KOH for 24 h and then rinsed in a few changes of water. The material was acidified in 5 % lactic acid for 24 h and then stained with 0.01 % aniline blue in lactic acid for 24 h. The entire procedure was carried out at room temperature.

The following parameters describing the intensity and effectiveness of the mycorrhization were recorded: mycorrhizal frequency (F%)—the ratio between root fragments colonized by AMF mycelium and the total number of root fragments analyzed; relative mycorrhizal root length (M%)—an estimate of the amount of root cortex that was mycorrhizal relative to the whole root system; the intensity of colonization within individual mycorrhizal roots (m%); relative arbuscular richness (A%)—arbuscule richness in the whole root system and arbuscule richness in root fragments where arbuscules were present (a%) (Trouvelot et al. 1986).

DSE colonization was identified on the basis of regularly septate hyphae, usually dark pigmented, with facultatively occurring sclerotia (Jumpponen 2001).

#### Data analysis

The two-way ANOVA was applied to examine the effect of a species and site on various variables, including the concentrations of metals in soils and plants, pH value and organic matter content, soil enzymes activity from root zone, root phosphatase activity, and mycorrhizal colonization indices. The Tukey’s test was used for multiple comparisons. To this end, based on the interactions of species and sites, six groups were distinguished which were further tested for significance of differences in the aforementioned variables. In relation to pH, the non-parametrical Kruskal-Wallis test followed by the Conover test for pairwise comparisons was used. The Pearson correlation test was employed to assess the significance of relationships between soil enzymes activity from root zone, root phosphatase activity, mycorrhizal colonization indices, and the properties of soils. All calculations were performed in R language and environment (R Core Team 2014).

## Results

Two-way ANOVA revealed that with regards to the interactions between species and sites, all cases of concentrations of metals in root zone turned out to be significant (Table 2). The contents of Mn, Zn, and Mg, Ca did differ among six groups based on species x site interactions (Table 2). The results are presented in details below.

**Table 2:** Effect of site, species on heavy metals in soils and in studied plants, pH, organic matter, soil enzymes, root phosphatase activity, and mycorrhizal colonization indices (two-way ANOVA and ^#^Kruskal-Wallis test)

Variable	Species			Site			Species x site		
	*d*	*F*	*p*	*d*	*F*	*p*	*d*	*F*	*p*
Cd	1	3.12	0.10259	2	3091.89	<0.00001	2	5.99	0.01569
Cu	1	391.89	<0.00001	2	474.47	<0.00001	2	255.66	<0.00001
Pb	1	19.84	0.0007872	2	7354.97	<0.00001	2	13.77	<0.00001
Fe	1	153.02	<0.00001	2	2630.26	<0.00001	2	80.77	<0.00001
Mn	1	145.64	<0.00001	2	7909.86	<0.00001	2	47.21	<0.00001
Zn	1	169.76	<0.00001	2	13120.02	<0.00001	2	97.15	<0.00001
Cd bioavailable	1	245.58	<0.00001	2	334.12	<0.00001	2	209.13	<0.00001
Pb bioavailable	1	1.76	0.2088	2	0.56	0.5829	2	0.29	0.7471
Mn bioavailable	1	150.24	<0.00001	2	179.33	<0.00001	2	36.62	<0.00001
Zn bioavailable	1	321.26	<0.00001	2	247.25	<0.00001	2	413.12	<0.00001
pH^#^	–	–	–	–	–	–	5	15.72	0.007687
Organic matter	1	13.76	0.002988	2	425.81	<0.00001	2	28.05	<0.0001
Cd shoot	1	227.54	<0.00001	2	836.23	<0.00001	2	106.03	<0.0001
Cu shoot	1	57.89	<0.00001	2	116.30	<0.00001	2	26.43	<0.0001
Pb shoot	1	0.044	0.8362	2	165.67	<0.00001	2	74.78	<0.00001
Fe shoot	1	38.80	<0.00001	2	157.10	<0.00001	2	4.31	0.03891
Mn shoot	1	1.64	0.2247	2	245.52	<0.00001	2	155.61	<0.00001
Zn shoot	1	43.20	<0.00001	2	359.95	<0.00001	2	35.53	<0.00001
Cd root	1	110.22	<0.00001	2	161.95	<0.00001	2	25.73	<0.0001
Cu root	1	23.56	0.0003959	2	51.34	<0.00001	2	39.87	<0.00001
Pb root	1	7.46	0.0182	2	1655.75	<0.00001	2	187.85	<0.00001
Fe root	1	100.19	<0.00001	2	1344.46	<0.00001	2	6.36	0.01308
Mn root	1	11.54	0.005301	2	11.54	0.005301	2	2.87	0.095584
Zn root	1	27.94	0.0001927	2	260.90	<0.00001	2	3.24	0.0749024
Mg shoot	1	192.64	<0.00001	2	80.89	<0.00001	2	74.59	<0.00001
Ca shoot	1	26.08	0.0002588	2	0.713	0.5099726	2	0.31	0.7383411
K shoot	1	0.17	0.688525	2	353.97	<0.00001	2	11.58	0.001583
Mg root	1	821.41	<0.00001	2	391.87	<0.00001	2	0.34	0.7217
Ca root	1	3.91	0.07138	2	24.91	<0.0001	2	0.62	0.55396
K root	1	85.67	<0.00001	2	114.46	<0.00001	2	20.23	0.0001432
Alkaline phosphatase	1	112.63	<0.00001	2	10333.65	<0.00001	2	594.17	<0.00001
Dehydrogenase	1	535.19	<0.00001	2	2563.93	<0.00001	2	238.03	<0.00001
Acid phosphatase	1	306.42	<0.00001	2	877.37	<0.00001	2	302.87	<0.00001
Urease	1	207.54	<0.00001	2	2266.67	<0.00001	2	157.15	<0.00001
Root acid phosphatase	1	368.42	<0.00001	2	206.4	<0.00001	2	43.53	<0.00001
*F*		0.0	1.0000	2	0.5	0.6186	2	1.5	0.2621
*M*		0.06	0.8093	2	1.73	0.2184	2	0.80	0.4700
*m*		0.07	0.7994	2	1.54	0.2531	2	0.94	0.4167
*a*		3.42	0.0891	2	1.73	0.2192	2	0.65	0.5394
*A*		1.7896	0.2058	2	2.6259	0.1133	2	0.2045	0.8179

### Root zone soil properties

The soil most contaminated with the examined metals was found in the mining activity area (D). Similar amounts of Cd were evaluated in root zone for both species of plantain (Table 3). Moreover in this stand, the highest Cu, Pb, and Zn content were characterized for the soil root zone of *P. lanceolata*, and for Fe and Mn, the highest concentrations in root zone soil were found for *P. major*. The bioavailable content of the examined metals was very low in comparison to the HNO_3_ extracted metal concentration, as listed in Table 3. pH values of the soils collected in the contaminated areas were above 7. Organic matter content was the highest in the soil samples from the site affected by mining activity (D).

**Table 3:** Heavy metals concentration, pH, and organic matter content in root zone soil of *P. lanceolata* and *P. major*

Species	Site	HNO_3_ extracted						Bioavailable			pH	Organic matter content
		Cd	Cu	Pb	Fe	Mn	Zn	Cd	Mn	Zn		
*P. lanceolata*	Sz	15.23 b	42.46 b	323.17 c	921.01 c	182.23 b	2723.84 c	1.03 a	0.73b	14.10 a	7.49 c	5.50 d
	D	69.28 a	122.65 a	809.24 a	2250.78 b	1247.76 d	28836.11 a	0.22 b	0.47 a	1.42 d	7.55 b	17.07 b
	J	0.79 d	8.31 d	34.48 e	343.74 e	48.41 e	342.10 e	0.25 c	1.26 cd	4.54 c	6.04 d	9.67 c
*P. major*	Sz	10.15 c	28.86 c	293.42 d	848.31 c	273.22 c	1727.75 d	0.24 b	0.64 c	7.59 b	7.63 a	3.23 d
	D	69.86 a	29.28 c	758.31 b	2966.75 a	1477.58 a	24086.82 b	0.07 b	0.39 b	3.05 c	7.62 a	22.87 a
	J	1.25 d	7.63 d	47.47 e	641.16 d	61.53 e	348.04 e	0.04 c	0.31 d	0.50 e	6.26 d	11.03 c

The different letters denote significant differences between particular metal concentrations in the HNO_3_ extracted and bioavailable fraction, organic matter content and pH value (*p* < 0.05)

### Metal bioaccumulation in roots and shoots

Metal bioaccumulation was analyzed in shoots and in roots of *P. lanceolata* and *P. major* (Table 4). The highest Fe content in shoots and in roots were found in the plants collected in the mining activity area (D). The highest Zn contents in shoots and in roots were found in *P. lanceolata* in the site affected by smelting activity (Sz) and in *P. major* in the area affected by mining activity (D). The highest bioaccumulation of Cd in the shoots was noticed in *P. major*. Comparing the bioaccumulation ability of the two investigated species, higher concentrations of Cd, Fe, and Zn were evaluated in the roots of *P. lanceolata* than *P. major* (Table 4). The values of mobility ratio (MR) for all the metals in shoots from contaminated areas showed that their absorption from the soil was not considerable. The MR values in *P. lanceolata* in the area affected by smelting activity (Sz) and in the site affected by mining activity (D) were for Cd 0.71, 0.04; Cu 0.1, 0.08; Pb 0.15, 0.03, Mn 0.36; 0.03; Zn 0.28, 0.02. In *P. major*, the MR values were in the same contaminated sites as follows: Cd 1.36, 0.14; Cu 0.16, 0.10; Pb 0.1, 0.6, Fe 0.65, 0.27; Mn 0.16, 0.05; Zn 0.27, 0.03. It confirms the low mobility of metals in soils with pH value over 7. In addition, a value of MR higher than 1 was characteristic for Cd and Fe computed for roots of both species. Effective translocation (TF > 1) was not observed for the shoots of either species from the contaminated area.

**Table 4:** Heavy metals contents in shoots and roots of *Plantago lanceolata* and *P. major*

Species	Site	Cd		Cu		Pb		Fe		Mn		Zn		Mg		Ca		K	
		Shoot	Root	Shoot	Root	Shoot	Root	Shoot	Root	Shoot	Root	Shoot	Root	Shoot	Root	Shoot	Root	Shoot	Root
*P. lanceolata*	Sz	10.9 b	61.1 a	4.1 c	29.4 a	48.1 a	123.8 c	731.8 a	1654.2 c	66.4 a	111.7 b	757.3 a	1006.9 a	2770.3 d	3434 d	15,314 bc	7495 c	18,571 c	13,675 bc
	D	3.0 c	48.0 b	9.6 b	26.0 ab	23.9 b	186.1 b	836.8 a	2348.2 a	39.0 b	249.0 a	630.3 b	980.1 ab	4126.5 b	4234 c	17,560 abc	11,640 ab	33,426 a	12,631 cd
	J	2.0 cd	4.9 d	13.0 a	18.8 b	12.8 c	26.2 e	432.8 b	570.9 e	27.8 c	62.5 cd	207.5 d	460.3 c	2627.8 d	4742 e	14,700 c	8561 bc	16,476 cd	9442 e
*P. major*	Sz	13.9 a	24.9 c	4.6 c	31.1 a	29.8 b	75.4 d	550.8 b	1306.1 d	44.6 b	79.0 bc	467.2 c	853.5 b	3433.6 c	3911 b	27,885 a	8456 bc	21,966 b	14,113 b
	D	10.1 b	26.1 c	3.1 c	4.1 c	47.5 a	276.0 a	790.8 a	2029.7 b	73.4 a	248.9 a	687.7ab	938.4 ab	4233.5 b	4548 a	26,682 ab	13,641 a	31,248 a	16,842 a
	J	1.5 d	2.3 d	10.0 b	20.8 b	6.5 c	9.7 e	246.5 c	437.5 e	20.4 c	28.9 d	107.9 e	278.3 d	4741.9 a	2710 c	24,009 abc	9019 ca	14,604 d	11,534 d

The different letters denote significant differences between the particular metal concentrations separately in shoots and roots of investigated plants (*p* < 0.05)

### Soil enzyme activities

**Fig. 1:**
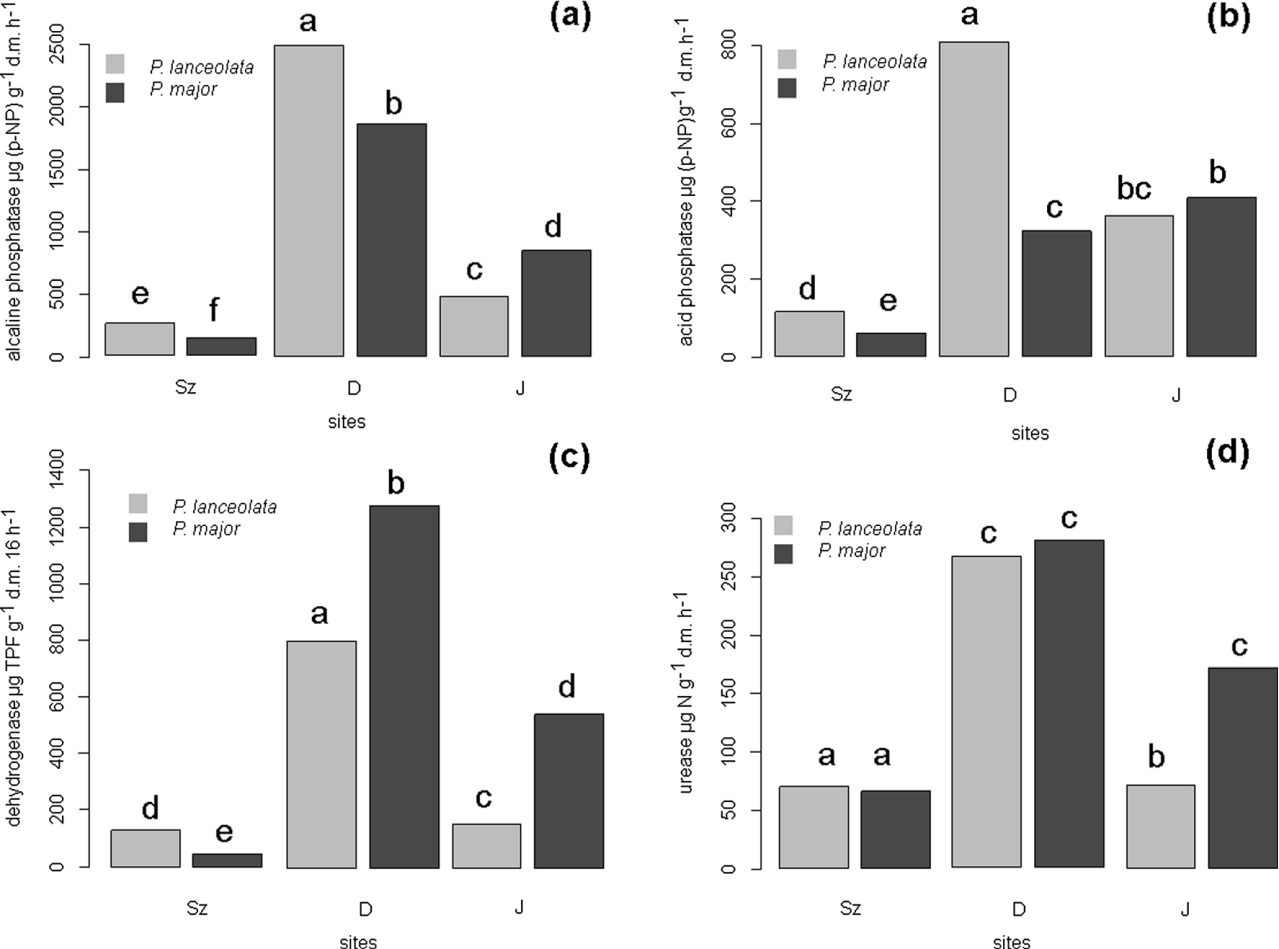
(a–d) Soil enzyme activity in root zone of *Plantago* species. **a** Alkaline phosphatase, **b** acid phosphatase, **c** dehydrogenase, **d** urease. The different letters denote significant differences between enzymes activity (*p* < 0.05)

Generally, root zone soils of both species collected in the site impacted by mining activity (D) were characterized by the highest enzyme activity (Fig. 1a–d). Soils from the root zone of *P. lanceolata* were characterized by the highest acid and alkaline phosphatase. In turn, the highest dehydrogenase and similar urease activity was observed in root zone soil samples of *P. major* in the same area. A lower activity of soil phosphatases, dehydrogenase as well as urease was found in the area impacted by smelting activity (Sz) in root zone soils of both species in comparison to soil from the uncontaminated control area.

To compare the heavy metal effects between the different contaminated soils (mining and smelting activity), an enzyme activity change ratio (ACR) was investigated. ACR depicts the relative toxicity of investigated soil to enzyme activity. A positive ACR in the area impacted by mining activity (D) denoted that enzyme activity was enhanced, and a negative ACR in the area impacted by smelting activity (Sz) denoted that enzyme activity was restricted (Table 5). ACR for the investigated soil enzymes varied from −67.6 to 424.4 for *P. lanceolata* root zone soil and from −85.6 to 136.9 for *P. major* root zone soil.

**Table 5:** Multiple comparison of enzyme activity change ratios (ACR) [%] in the root and root zone soil of *P. lanceolata* and *P. major*

Species	Contaminated site	Alkaline phosphatase	Acid phosphatase	Dehydrogenase	Urease	Root acid phosphatase
*P. lanceolata*	Sz	−47.06 c	−67.63 c	−17.23 c	−0.98 c	−25.74 b
*P. major*	Sz	−83.87 d	−85.60 d	−91.85 d	−61.83 d	−42.41 c
*P. lanceolata*	D	424.41 a	126.53 a	422.88 a	277.76 a	1.93 a
*P. major*	D	120.49 b	−20.81 b	136.91 b	63.46 b	7.71 a

There is no significant difference among ratios followed by the same letter (*p* < 0.05)

Positive correlation coefficients were obtained between the examined soil enzymes and metals extracted with HNO_3_ (Table 6). Also, positive correlation coefficients were obtained between investigated enzymes activity and bioavailable Mn content in soil from the root zone of *P. lanceolata*. Soil enzymes activity was positively correlated to organic matter content.

**Table 6:** Correlation coefficients between soil enzymes activity from root zone of *P. major* and *P. lanceolata*, root phosphatase activity, mycorrhizal colonization indices, and soil properties

	Acid phosphatase	Alkaline phosphatase	Dehydrogenase	Urease	Root acid phosphatase	F	M	m	a	A
*Plantago major*										
Cd	0.17	0.86*	0.86*	0.80*	0.52	−0.51	−0.49	−0.45	−0.09	−0.35
Cu	−0.67*	0.12	0.13	0.03	−0.35	−0.26	−0.77*	−0.77*	−0.27	−0.65
Pb	−0.06	0.72*	0.73*	0.65	0.31	−0.47	−0.61	−0.57	−0.14	−0.45
Fe	0.20	0.88*	0.88*	0.83*	0.55	−0.52	−0.48	−0.43	−0.06	−0.32
Mn	0.15	0.85*	0.85*	0.79*	0.50	−0.50	−0.50	−0.45	−0.10	−0.35
Zn	0.23	0.89*	0.89*	0.84*	0.57	−0.51	−0.46	−0.41	−0.09	−0.33
Cdbio	−0.59	0.21	0.22	0.11	−0.26	−0.27	−0.63	−0.62	−0.21	−0.52
Mnbio	−0.21	0.57	0.58	0.50	0.11	−0.27	−0.74*	−0.73*	−0.13	−0.52
Znbio	0.17	0.86*	0.86*	0.80*	0.51	−0.50	−0.51	−0.47	−0.09	−0.36
pH	−0.69*	0.10	0.11	0.00	−0.37	−0.27	−0.73*	−0.72*	−0.20	−0.57
Organic matter content	0.64	0.99*	1.00*	0.99*	0.85*	−0.41	−0.17	−0.13	−0.02	−0.10
*Plantago lanceolata*										
Cd	0.85*	0.96*	0.97*	0.98*	0.37	0.12	−0.26	−0.28	0.03	−0.11
Cu	0.79*	0.92*	0.94*	0.95*	0.28	0.11	−0.32	−0.33	−0.08	−0.22
Pb	0.74*	0.89*	0.91*	0.93*	0.20	0.06	−0.24	−0.25	−0.10	−0.20
Fe	0.79*	0.92*	0.94*	0.95*	0.28	0.09	−0.26	−0.26	−0.03	−0.16
Mn	0.90*	0.98*	0.99*	0.99*	0.46	0.19	−0.27	−0.29	0.10	−0.06
Zn	0.91*	0.99*	0.99*	1.00*	0.48	0.22	−0.28	−0.30	0.12	−0.04
Cdbio	−0.63	−0.42	−0.37	−0.34	−0.97*	−0.43	0.12	0.18	−0.71*	−0.50
Mnbio	0.72*	0.87*	0.90*	0.91*	0.17	0.11	−0.26	−0.27	−0.16	−0.26
Znbio	−0.67*	−0.47	−0.42	−0.39	−0.97*	−0.40	0.11	0.16	−0.71*	−0.51
pH	0.21	0.45	0.50	0.53	−0.41	−0.23	−0.13	−0.10	−0.49	−0.46
Organic matter content	0.99*	0.96*	0.94*	0.93*	0.80*	0.39	−0.26	−0.31	0.46	0.23

*Coefficient are statistically significant at *p* < 0.05

### Root phosphatase activity

**Fig. 2:**
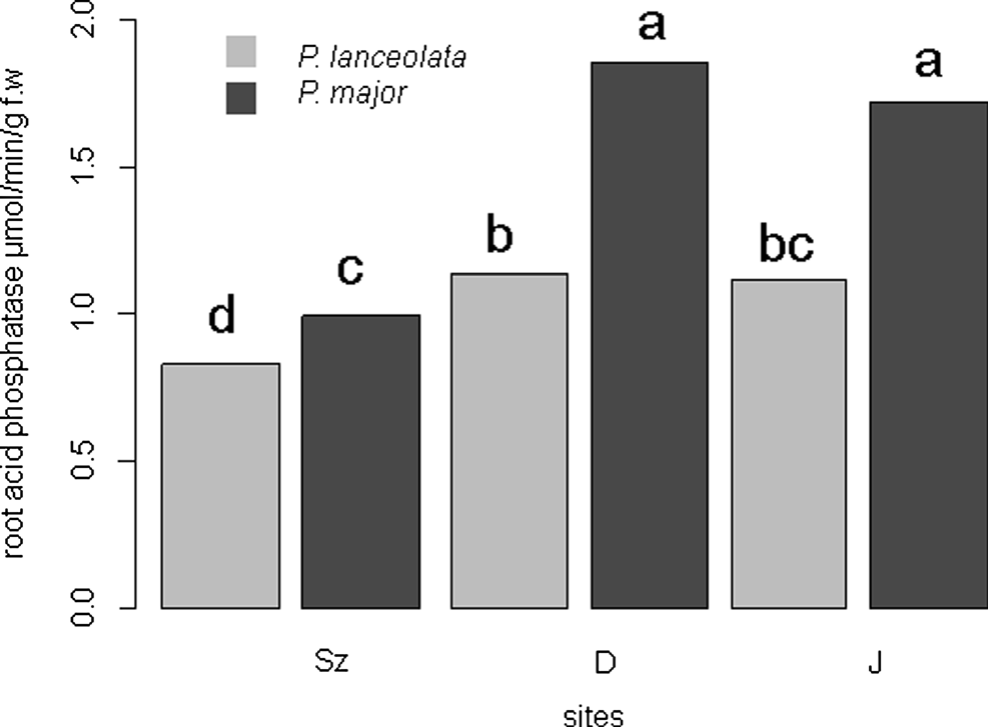
Root acid phosphatase activity in *Plantago* species. The different letters denote significant differences between enzymes activity (*p* < 0.05)

The root acid phosphatase activity was higher in *P. major* plants collected in the area disturbed by mining activity (D) comparing to the plants collected in area disturbed by smelting activity (Sz) and similar to plants from the control area (Fig. 2). ACR for root acid phosphatase varied from −25.7 to 1.93 for *P. lanceolata* and from 42.4 to 7.71 for *P. major* (Table 5). Root acid phosphatase was positively correlated to organic matter in the soil (Table 6). Negative correlation coefficients were obtained between root acid phosphatase in *P. lanceolata* and soil bioavailable Cd and Zn concentrations (Table 6).

### Mycorrhizal colonization

**Fig. 3:**
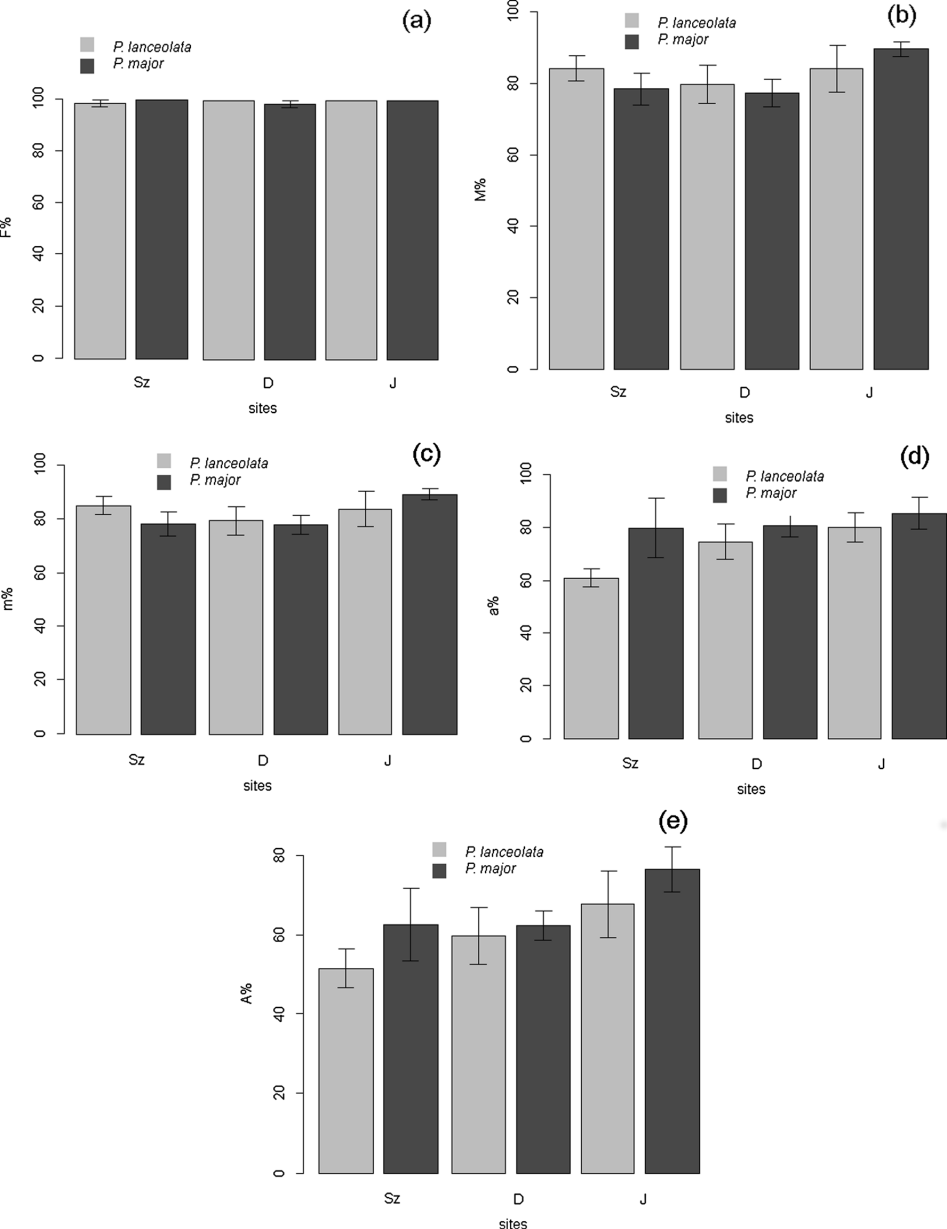
Mycorrhizal colonization in *Plantago* species roots. **a** Mycorrhizal frequency (F %), **b** relative mycorrhizal root (M %), **c** the intensity of colonization within individual mycorrhizal roots (m %), **d** arbuscule richness in root fragments, where the arbuscules were present (a %), **e** the relative arbuscular richness (A %)

Arbuscular mycorrhizae with arbuscules, which are the structural and functional criterion of symbiosis, were found in the roots of both analyzed *Plantago* species in all studied stands. In addition to the arbuscules, vesicles and coils were also present. All of the plants examined showed the *Arum*-type morphotype in which AM fungi most often colonize the inner cortex cells and arbuscules are formed terminally. In roots of both plantains, coarse AM fungi (hyphae diameter above 2 μm) were mostly found. Fine endophyte (*Glomus tenue*) was observed only in *P. lanceolata* roots from Dąbrowa Górnicza. The roots in both *Plantago* species were almost fully colonized by the AM mycelium (F% ranged from 98 to 100 %) (Fig. 3a). Roots of plantains from contaminated (D, Sz) and non-contaminated (J) areas showed no statistically significant differences in mycorrhizal colonization. However, the lowest root colonization (M%, m%) was found in *P. major* and *P. lanceolata* in the area affected by mining activity (D) (Fig. 3b, c). In terms of arbuscule richness, the highest values of both measures of root arbuscule occurrence (A% and a%) in both investigated species were high in all the investigated sites. Arbuscule abundance in the whole root system (A%) exceeded 60 %, whereas arbuscule richness of the colonized root section (a%) was over 50 %. The lowest arbuscule occurrence was found in Szopienice, the area affected by smelting activity (Fig. 3d, e).

The richness of mycorrhizal structures in the roots varied only slightly between plantains species. In the case of *P. lanceolata*, the intensity of root colonization (M%, m%) was higher in areas affected by mining and smelting activity compared to *P. major*, which was higher in the uncontaminated site. In contrast, average arbuscule abundance was higher in *P. major* in all studied stands when compared to *P. lanceolata* (Fig. 3).

All mycorrhizal indices were negatively correlated with the bioavailable contents of Zn, Cd, and Mn and with other metal concentrations in the soil (extracted with HNO_3_), while only statistically significant in terms of the richness of arbuscules in mycorrhizal root fragments (a%) in *P. lanceolata* roots and bioavailable Zn and Cd soil content, as well as between the intensity of root colonization (M%, m%) in roots of *P. major* and Cu and bioavailable Mn concentration in the soil (Table 6). Cd, Pb, and Zn concentrations in roots and shoots were negatively correlated with mycorrhizal colonization indices in roots of both investigated plantains (however, not all correlation coefficients were statistically significantly).

### DSE colonization

DSE were found in both investigated plant species. However, they were not present in all the root samples. The mycelium was brownish and did not stain with aniline blue but remained brownish. DSE were observed in the cortex together with AMF but mainly in root fragments where arbuscules were absent. Only single hyphae, accompanied sporadically by sclerotia, were found in the rhizodermis and outer cortical cells. DSE were not observed in the roots of *P. lanceolata* or *P. major* from Szopienice, the site affected by smelting activity.

## Discussion

In our work, we present a detailed report of soil enzymatic activities in the rhizosphere and arbuscular mycorrhizal colonization in roots of *P. lanceolata* and *P. major* in the areas affected by mining and smelting activity in southern Poland. Such field studies are needed for the verification of results obtained under controlled conditions.

Metal concentrations in the investigated areas (D and Sz) definitely exceeded the average concentrations and limit values in the soil. Zn concentration in the soil root zone of *P. lanceolata* and *P. major* in the non-contaminated site (J) slightly exceeded the permissible concentration. Under natural conditions, soil levels of Zn usually fall in the range 10–300 mg kg^−1^. The mean Pb concentration for surface soils on a world scale is estimated at 25 mg kg^−1^ and the average content of Cd in soils between 0.06 and 1.1 mg kg^−1^ (Kabata-Pendias 2001). However, as reported in the Regulation by the Polish Minister of the Environment (2002), the limit values (permissible concentrations) of heavy metal content in soils are Zn—300 mg kg^−1^, Pb—100 mg kg^−1^, and Cd—4 mg kg^−1^.

In our study, *Plantago* species exhibited higher metal concentrations in the roots than the corresponding shoot sample. Similar results were obtained for plants occurring on toxic mine tailings in Chenzhou City, Hunan Province, and a control site in Hong Kong in a work by Leung et al. (2007). Also, very low values of mobility ratio (syn. bioconcentration factor below 0.006, 0.002, and 0.02 for Zn, Pb, and Cd, respectively) in *P. lanceolata* were obtained by Wójcik et al. (2014) investigating waste deposits (Zn-Pb) in Brzeziny Śląskie and Bolesław (southern Poland). In that study, *P. lanceolata* accumulated in shoots the following ranges of the Zn, Pb, Cd, Cu, respectively, 80.7–444; 3.4–38.2, 0.3–7.1, and 5.1–14.5 mg kg^−1^ (Wójcik et al. 2014). The accumulated amounts of the above-mentioned metals in our study were higher for Zn, Pb, Cd and similar for Cu. Plants selected for phytostabilization should be characterized by a low accumulation of metals in the above ground parts, restricted metal translocation from roots to shoots, dense canopies, and root system with a fast growth and high tolerance to metal contaminants and other adverse site conditions (Ernst 2005; Wójcik et al. 2014). Wójcik et al. (2014) suggested some plants for such protective treatment, with the following Zn, Cd, and Pb accumulation in the shoots of *Calamagrostis epigejos* (36–91; 0.2–3; 6–11 mg kg^−1^), *Carex hirta* (72–275; 0.6–7,18; 2–19 mg kg^−1^), *Dianthus carthusianorum* (152–463.4; 3.5–10.5; 3–11 mg kg^−1^), *Thymus pulegioides* (109–445; 0.3–1.2; 4.8–40 mg kg^–1^); *Scabiosa ochroleuca* (108–569; 0.3–3; 3–55), and *Trifolium repens* (90–207.6, 0.2–0.9, 3 mg kg^−1^). Metal accumulation in the two *Plantago* species in our investigation was inside the ranges mentioned above. Moreover, in our extensive study, we found some direct positive correlations between the amount of heavy metals in the soil and bioaccumulation in roots and shoots of the plants. In addition, a higher correlation coefficient between metal accumulation in soil and in roots was observed, especially for Pb. All these characteristics of *P. lanceolata* and *P. major*, in addition to high mycorrhizal colonization, as well as tolerance to metal contaminants and other adverse site conditions connected with antioxidant defense (Nadgórska-Socha et al. 2013), make them potentially effective phytostabilizers.

Root phosphatases catalyze the hydrolysis of various phosphate esters increasing P available to plants and thus enhancing plant uptake (Carrasco et al. 2011). The soils in the investigated areas were characterized by small amounts of P extracted with HNO_3_ (D—3.3, Sz—185, J—267.5 mg kg^−1^) (Nadgórska-Socha et al. 2015). Generally, in our study, we found a positive correlation coefficient between mycorrhizal colonization and root phosphatase activity, although not statistically significant. In a study by Carrasco et al. 2011, mycorrhizal and fungal inoculation enhanced the phosphatase in the root of *Coronilla juncea* cultivated in soil from mine tailings. Increased root phosphatase activity under heavy metal stress is one of the possible processes involved in detoxification and resistance (Carrasco et al. 2011). In our study, decreased or the same activity of root phosphatase in plantains from contaminated areas was observed in comparison to the activity in the control site. Therefore, heavy metal phosphatase activity inhibition seems to depend on the plant species and metal concentration.

Soil enzymes activity is often used as an indicator of the functioning of soil ecosystems as well as anthropogenic input in soil environments. Moreover, enzyme activity can be used to show the effectiveness of soil rehabilitation treatments or to reflect soil quality that was destroyed by industrial processes (Ciarkowska et al. 2014). Ciarkowska et al. 2014 show that a high organic matter content as well as neutral pH transform toxic metals into biologically inactive forms. Those findings correspond with our results especially for the site affected by mining activity (D) (highest metal content, highest organic matter content and pH, with the highest soil enzyme activity). On the other hand, Niemeyer et al. (2012) indicate that the main negative effect on microbial indicators, and among these soil enzymes, seems to be due to the limitation of plant reestablishment that results in low amounts of organic matter inputs to the soil. Such limitation of plant reestablishment was found in the area affected by smelting activity (Sz). This site is an example of an area on which infertility of the substratum (low organic matter), due to high bioavailable metal content, spontaneous revegetation is a very long process. The vegetation of this place is still in the early stage of succession. A higher organic matter content in the soil, as we found in the post mining area (D), supported greater soil enzyme activity not only by acting as sources of C for the soil microbial community but also due to the chelating effect protecting microorganisms and soil enzymes from excessive levels of metals in the soil (Lejon et al. 2010; Niemeyer et al. 2012). Tripathy et al. 2014 also found that all investigated soil enzymes were positively correlated with organic carbon, which indicates that enzyme activity is associated with organic fraction. Moreover, in the experiment of Patel and Patra (2014), the increased activities of dehydrogenase, acid, and alkaline phosphatase in tannery sludge rich in heavy metals were presumably due to the high presence of organic matter. Water-soluble and exchangeable metal forms are generally more toxic than other forms because they can easily be released into water. The activity of soil microorganisms decreases as the levels of water-soluble and exchangeable metals increase (Tripathy et al. 2014). Accordingly, a negative correlation was found between bioavailable Zn and acid phosphatase activity in *P. lanceolata* root zone soil. Also, Wang et al. (2007) found negative correlations between soil phosphatase activity and NH_4_NO_3_ extractable heavy metals. Moreover, they suggested that soil microorganism activity and community composition could be predicted using the availability of Cu and Zn. The negative ACRs values were computed for the area affected by smelting activity (Sz), confirming inhibition of soil enzyme activity. A similar statement on the usage of ACR in soil enzyme activity investigations was made for soil contaminated with Pb, Cd, and As, in Xian et al. (2015). Generally, in our study in the soil root zone of *P. lanceolata*, phosphatase activity was higher in comparison to *P. major* in contaminated areas. This is in agreement with findings that different plant species can associate with microbial communities with unique characteristics probably due to differences in the amount and quality of root exudates (Yang et al. 2007). Moreover, it is also suggested that the coexistence of more plant species may alleviate Pb and Cd impacting on the activity of enzymes (Yang et al. 2010).

The fact that mycorrhizal colonization occurred in all of our in situ observations suggests a metal tolerance of local AM fungi. A well-developed mycorrhizal symbiosis may enhance the survival of plants in areas contaminated with heavy metals by better nutrient acquisition, water relations, pathogenic resistance, phytohormone production, and contribution to soil aggregation (Smith and Read 2008). Our study, a comparison of sites affected by smelting and mining activity and an uncontaminated control area, revealed no statistically significant differences in mycorrhizal colonization and arbuscule occurrence between *P. lanceolata* and *P. major* roots. This is in accordance with the findings of other authors (Ietswaart et al. 1992; Weissenhorn et al. 1995a, b; Rozpądek et al. 2014). There was no significant difference in mycorrhizal root colonization between populations of *Agrostis capillaris* growing on a sandy soil polluted by a smelter compared to limestone-derived clay with or without metals of natural origin (Ietswaart et al. 1992). High levels of mycorrhizal colonization were also observed in agricultural soils contaminated with metals from a smelter and sludge amendments (Weissenhorn et al. 1995a, b). A recent study in Poland demonstrated no statistically significant differences in mycorrhizal colonization (F% 100, M% 93–95, A% 60–88) in chicory roots inoculated with *Rhizophagus irregularis* growing under the presence of toxic metals from industrial waste substratum from ZG Trzebionka and from non-polluted substrata (Rozpądek et al. 2014). The roots were in both cases almost fully colonized by the mycelium. On the other hand, metal-tolerant *Oxalis acetosella* colonizing forest soils treated with Cd, Zn, and Pb containing industrial dusts showed even higher AM colonization than non-treated soils (Turnau et al. 1996).

Our results demonstrated DSE colonization in both *Plantago* species in an area affected by mining activity (D) and in an uncontaminated stand (J). DSE is known to frequently colonize the roots of plants growing in heavy metal contaminated soil (Gucwa-Przepióra et al. 2013; Likar and Regvar 2013; Xu et al. 2015). Recently, Affholder et al. (2014) demonstrated that DSE may alleviate the toxicity of excess metal ions in host plants similarly to mycorrhizal fungi. However, in our investigation, DSE were not observed in the roots of *P. lanceolata* or *P. major* from a place affected by smelting activity (Sz), where the highest metal bioavailability was found. These results suggest that excessive concentrations of metals may reduce DSE occurrence in roots.

## Conclusions

The area affected by smelting activity had a lower level of investigated soil enzymes, as well as a higher bioavailability of metals in the root zone soil of the *Plantago* species compared to the control site. Limitation of plant cover reestablishment, low organic matter in the soil as well as high bioavailable metal concentrations causes a decrease in enzyme activities. High organic matter concentration in the soil and a neutral or alkaline pH transform metals into biologically inactive forms. In this study, it was observed at the site affected by mining activity, where the highest soil enzymes activity was found.

Mycorrhizal colonization in both species in the contaminated areas was similar to the uncontaminated site. The lowest arbuscule occurrence was found in the area affected by smelting activity where the highest metal bioavailability was found. Moreover, DSE were not observed in the roots of the *Plantago* species in that stand, too. In pot experiments (in restricted conditions) conducted by other investigators, *P. lanceolata* AM indices were shown to be good indicators of soil heavy metal contamination. However, in our field study, such clear relations were not confirmed; this discrepancy shows that the problem needs further investigations.

The investigated *Plantago* species exhibited the excluder strategy and accumulated higher metals content in roots than in shoots. The accumulation in their shoots was comparable to other plants suggested as phytostabilizers in literature. The high tolerance to metals may be associated with a well-functioning mycorrhizal symbiosis observed in conditions that are highly injurious for plants. A decrease in the mycorrhizal colonization in contaminated sites was not observed in both plantains species. Due to the low accumulation of metals in shoots, as well as a high tolerance to metal contaminants, the selected *Plantago* species may be applied in the phytostabilization of heavy metal contaminated areas.

The combination of diverse monitoring approaches and the biological and physico-chemical methods in our study (assessment of enzyme activity, mycorrhizal colonization, and the chemical and physical properties of soils) proved to be sensitive to differences between sites and between *Plantago* species. This observation is very important in terms of land reclamation.
